# Anti-carbamylated Protein Antibodies Positivity in Rheumatoid Arthritis and Its Association With Rheumatoid Factor and Anti-cyclic Citrullinated Protein Antibodies

**DOI:** 10.7759/cureus.63652

**Published:** 2024-07-02

**Authors:** Dhanush Balaji, Kavitha Mohanasundaram, Karpaka Vinayakam Gopalakrishnan, Prasanna Karthik Suthakaran

**Affiliations:** 1 Internal Medicine, Saveetha Medical College Hospital, Saveetha Institute of Medical and Technical Sciences, Saveetha University, Chennai, IND; 2 Rheumatology, Saveetha Medical College Hospital, Saveetha Institute of Medical and Technical Sciences, Saveetha University, Chennai, IND

**Keywords:** anti-ccp antibodies, anti-carp antibodies, ra factor, autoimmune disease, rheumatoid arthriitis

## Abstract

Background

Rheumatoid arthritis (RA) is a widespread autoimmune disease affecting millions of people worldwide. The current markers include anti-cyclic citrullinated peptide (anti-CCP) antibodies and rheumatoid factor (RF), which are nonspecific and elevated in various conditions and do not have a prognostic value. They are also elevated in the later stages of the disease. Anti-carbamylated protein (anti-CarP) antibodies have been reported to be associated with joint damage in RA. Therefore, this study aimed to evaluate the sensitivity and specificity of anti-CarP antibodies in individuals with RA and their relationship with inflammatory markers such as C-reactive protein (CRP) and erythrocyte sedimentation rate (ESR).

Methods

This was a cross-sectional case-control study conducted from April 2020 to March 2021 at the Saveetha Medical College, Chennai, India. The age makeup of the three groups was evaluated: Group 1 comprised anti-CCP and RF-positive patients; Group 2 comprised anti-CCP and RF-negative patients; and Group 3 was the control group, which comprised healthy volunteers. Patient samples, including blood and serum, have been utilized to conduct various assessments aimed at evaluating biomarkers such as CRP, ESR, RF, and autoantibodies like anti-CCP and anti-CarP.

Results

This study examined the role of various autoantibodies and biomarkers in RA across three distinct groups. Group 1 predominantly consisted of middle-aged individuals, and women constituted the majority in both Group 1 and Group 2, consistent with higher RA prevalence among females. In Group 1, 65.7% of patients tested positive for anti-CarP, while in Group 2, 48.6% tested positive even when RF and anti-CCP antibodies were absent. This suggests a potential diagnostic role for anti-CarP antibodies in identifying RA patients early. CRP and ESR levels were significantly elevated in RA patients (Groups 1 and 2) compared to healthy controls (Group 3), indicating higher inflammatory activity in affected individuals. We also observed that anti-CarP antibodies had a specificity of 69.1% and a sensitivity of 78.2%. Positive correlations between the diagnosis of RA and anti-CarP antibody positivity were observed across the groups and correlated well with the inflammatory markers and signs such as joint damage. The data were found to be statistically significant.

Conclusions

Our study showed a significant correlation between joint damage and CRP levels and a positive correlation between anti-CarP antibodies and ESR and CRP values. These findings suggest that anti-CarP antibodies can offer certain advantages over RF and anti-CCP antibodies in RA diagnosis due to their early detection potential, higher specificity, complementary diagnostic role, and predictive value for disease severity.

## Introduction

Rheumatoid arthritis (RA) is a prevalent autoimmune disorder affecting millions globally, characterized by systemic inflammation leading to joint pain and dysfunction. According to the 2010 Global Burden of Disease (GBD) assessment, RA prevalence was estimated at 0.24% [1,2], affecting approximately 1% of the adult population, with middle-aged women comprising one-third of affected individuals [3]. RA entails inflammation, swollen joints, joint degeneration, disability, and potential mortality [4,5]. Diagnosis typically relies on detecting specific antibodies in serum and synovial fluid, which is pivotal for disease prognosis and management [6]. Historically, autoantibodies such as anti-cyclic citrullinated peptide (anti-CCP) and rheumatoid factor (RF) have been vital diagnostic markers [1]. These autoantibodies are found in 70%-80% of RA cases, though they are not exclusive to RA and are also present in other immune-related disorders [7-9]. Anti-CCP offers higher diagnostic specificity than RF, being positive in approximately two-thirds of RA patients [10,11], and is associated with disease severity and progression [12,13]. Recent research has identified a distinct autoantibody system, separate from RA factors and anti-CCP antibodies, targeting carbamylated proteins (anti-CarP antibodies), detected in 36%-45% of RA patients [14,15]. Anti-CarP antibodies often precede RA symptoms by several years, indicating their role in chronic disease development [16]. Moreover, patients positive for anti-CCP antibodies often exhibit anti-CarP antibodies, suggesting their potential as diagnostic markers [17]. Thus, this study aimed to evaluate the sensitivity and specificity of anti-CarP antibodies in RA patients.

## Materials and methods

This research was conducted at the Department of General Medicine and the Department of Rheumatology, Saveetha Medical College and Hospital, located in Chennai, Tamil Nadu, India. This was a cross-sectional case-control study with a duration of 12 months, from April 2020 to March 2021. The Institutional Ethics Committee approved the study. This research was conducted on RA patients diagnosed and treated at the institution's Department of Rheumatology. The following criteria were used to include participants in the study: being at least 18 years old, having RA according to the 2010 American College of Rheumatology/European League Against Rheumatism (ACR/EULAR) criteria, and willingness to participate [1]. The following criteria were used to exclude participants from the study: systemic lupus erythematosus, mixed connective tissue disorders (overlap syndrome), Sjogren’s syndrome, psoriatic arthritis, sarcoidosis, osteoarthritis, chronic kidney disease, and chronic liver disease. The sample size for the study was determined using a nonprobability convenience sampling method. It was based on previous studies' sensitivity and specificity of autoantibodies for RA, a 20% margin of error, and a 99% confidence interval. A total of 90 participants were included in this study.

In this study, eligible participants were individuals diagnosed with RA who tested positive for RF and anti-CCP, as well as those who tested negative for both biomarkers. We aimed to have an equal number of participants in each group to evaluate anti-CarP antibody positivity in seronegative arthritis. The inclusion and exclusion criteria were applied to all subjects. Upon enrollment, eligible candidates were interviewed for their sociodemographic information, and a detailed medical history was obtained, followed by a physical examination. All collected data were documented on the subject's designated case form. The participants were then classified and enrolled in accordance with the 2010 ACR/EULAR criteria. By employing patient samples such as blood and serum, various assessments have been conducted to evaluate C-reactive protein (CRP), erythrocyte sedimentation rate (ESR), RF, and autoantibodies. CRP levels were determined using the chemiluminescent immunoassay method, with a negative result indicated by a range of <10 mg/L and a positive result of >10 mg/L. To evaluate ESR, measurements were taken using an automated analyzer, with normal ranges for males at 0-18 mm/hour and for females at 0-20 mm/hour. RF was assessed through nephelometry, with negative results indicated by a range of <20 IU/L and positive results of >20 IU/L. Anti-CCP and anti-CarP antibodies were quantitatively measured using enzyme-linked immunosorbent assay kits from the Fine Test (Boulder, Colorado), exhibiting high sensitivity and specificity for antibody detection. No significant cross-reactivity or interference between autoantibodies and analogs was observed. To ensure optimal performance and minimize external influences, strict control measures were implemented under laboratory conditions, including room temperature, air humidity, and incubator temperature. The anti-CarP antibody range used for testing was <4.25, with negative results indicated by a value within this range and positive results by a value greater than 4.25. Similarly, the anti-CCP range used was <25 U/mL for negative results and >25 U/mL for positive results, respectively.

The presence of hand deformities, such as swan neck deformity, boutonniere deformity, hitchhiker's thumb, rheumatoid nodules, and radiological changes, such as marginal erosions, joint space narrowing, subchondral cysts and erosions, joint destruction, magnetic resonance imaging (MRI) findings suggestive of synovitis, and joint effusions, were used to assess joint damage in subjects. Statistical analyses were conducted using the IBM SPSS Statistics for Windows, Version 20 (Released 2011; IBM Corp., Armonk, New York). The normal distribution of the data was tested using the Kolmogorov-Smirnov test, while Levene's test was used to assess the homogeneity of the variables. Variables without a normal distribution and equal variance were analyzed using Welch's ANOVA and Games-Howell post-hoc test between groups, and the results were expressed as mean, standard deviation, and significance. Qualitative data analysis was performed using the chi-squared test. A p-value of less than .05 was considered to indicate statistical significance.

## Results

**Figure 1:**
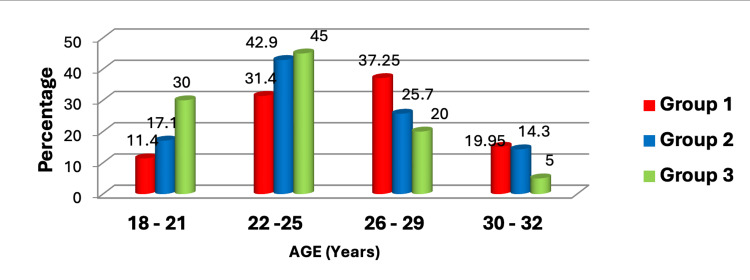
Distribution of age among rheumatoid arthritis patients and healthy controls. This figure represents the data in the form of percentages (N1=35, N2=35, N3=20). A p-value less than 0.05 is considered statistically significant; here, the p-value was found to be 0.374 by the Fisher exact test.

The age distribution of three distinct groups was analyzed: Group 1 comprised individuals who were positive for both anti-CCP and RF; Group 2 included those negative for both anti-CCP and RF; and Group 3 comprised healthy controls. The majority of subjects in Group 1 fell within the 41-50 age bracket, which aligns with typical demographics for middle-aged individuals. Previous research has indicated a higher incidence of RA within this age range. Interestingly, despite testing negative for both RF and anti-CCP antibodies, Group 2 exhibited the highest proportion of participants aged 31-40 (Figure 1).

**Figure 2:**
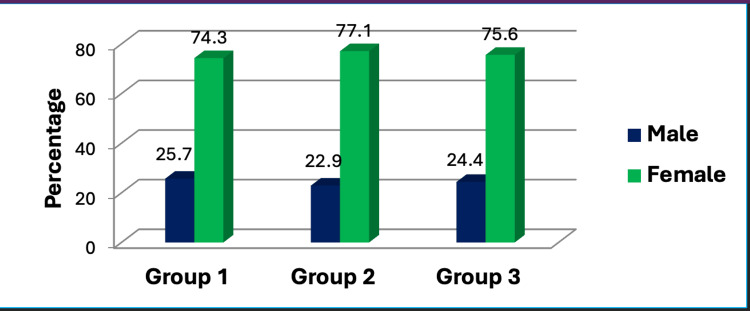
Sex distribution among patients with rheumatoid arthritis and healthy controls using the chi-square test. This figure represents the data in the form of percentages (N1=35, N2=35, N3=20). A p-value less than 0.05 is considered statistically significant; here, the p-value was found to be 0.960 by Pearson's chi-square test.

Gender disparities among the study participants were examined. Notably, women constituted the predominant gender in our sample, comprising 74% of Group 1 and approximately 77% of Group 2. Consistent with prior investigations, our study unveiled that approximately two-thirds of female subjects were afflicted by RA compared to their male counterparts (Figure 2).

The association between anti-CarP antibodies and RA was also investigated. In Group 1, 23 of the 35 patients tested positive for all three autoantibodies, accounting for 65.7% of the Group 1 population. In Group 2, 17 of the 35 individuals tested positive for anti-CarP antibodies only, despite negative results for the other two. A significant number of RA patients in Group 2 tested positive for anti-CarP antibodies despite testing negative for both RF and anti-CCP antibodies (Table 1).

**Table 1:** Association of anti-carbamylated protein antibodies (biomarkers) among rheumatoid arthritis patients and healthy controls by using the chi-square test. ***This table represents the data in the form of percentages (N1=35, N2=35, N3=20). A p-value less than 0.05 is considered statistically significant; here, the p-value was found to be 0.000, making it significant. Anti-CarP: anti-carbamylated protein; RA: rheumatoid arthritis.

Anti-CarP (Biomarker)	RA Patients			
	Group 1 N (%)	Group 2 N (%)	Group 3 N (%)	Total N (%)
Positive	23 (65.7)	17 (48.6)	02 (10)	42 (46.7)
Negative	12 (34.3)	18 (51.4)	18 (90)	48 (53.3)
Total	35 (100)	35 (100)	20 (100)	90 (100)
Pearson’s chi-square test value=15.957, p=0.000***				

Table 2 illustrates the relationship between elevated CRP levels and RA in both patient groups and healthy controls. Notably, individuals from Group 2 who tested negative for traditional antibodies exhibited a higher incidence of elevated CRP levels. In Group 2, approximately 80% of the subjects tested positive for CRP, while only 37% of Group 1 showed positive CRP values (Table 2).

**Table 2:** Association between C-reactive protein (CRP) levels in rheumatoid arthritis patients and healthy controls using the chi-square test. ***This table represents the data in the form of percentages (N1=35, N2=35, N3=20). A p-value less than 0.05 is considered statistically significant; here, the p-value was found to be 0.000, which makes the data statistically significant. CRP: C-reactive protein; RA: rheumatoid arthritis.

CRP	RA Patients			
	Group 1 N (%)	Group 2 N (%)	Group 3 N (%)	Total N (%)
Positive	13 (37.1)	28 (80)	0	41 (45.6)
Negative	22 (62.9)	07 (20)	20 (100)	49 (54.4)
Total	35 (100)	35 (100)	20 (100)	90 (100)
Pearson’s chi-square test value=34.476, p=.000***				

**Figure 3:**
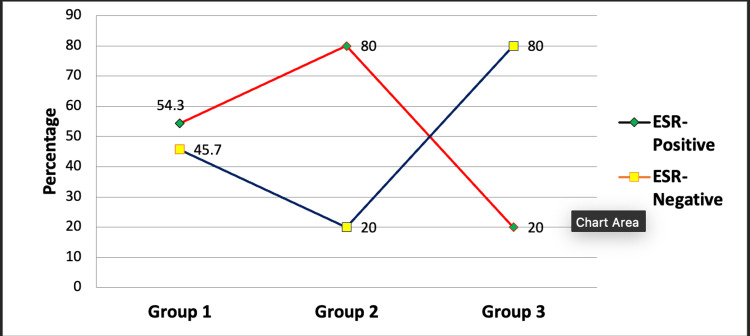
Association of erythrocyte sedimentation rate (ESR) between rheumatoid arthritis patients and healthy controls using the chi-square test. This figure represents the data in the form of percentages (N1=35, N2=35, N3=20). A p-value less than 0.05 is considered statistically significant; here, the p-value was found to be 0.000, which makes the data statistically significant. ESR: erythrocyte sedimentation rate.

Figure 3 shows the association between ESR in RA patients and healthy controls. ESR levels, like CRP, were greater in RA patients compared to healthy controls. Furthermore, almost 80% of those who tested negative for the two antibodies had a higher ESR, while 54.3% of those who were positive for both antibodies had an elevated ESR (Figure 3).

**Figure 4:**
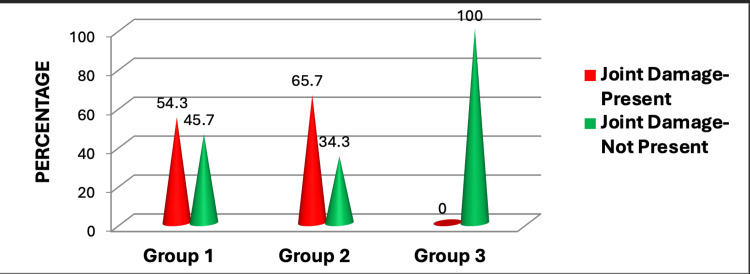
Association of joint damage between rheumatoid arthritis patients and healthy controls. This figure represents the data in the form of percentages (N1=35, N2=35, N3=20). A p-value less than 0.05 is considered statistically significant; here, the p-value was found to be 0.000, which makes the data statistically significant.

Figure 4 depicts the association between joint damage in different groups. Notably, Group 3, which included healthy controls, showed no joint injury. In line with previous research, Group 2 showed the most joint injury. Individuals who tested negative for RF and anti-CCP antibodies may have had delayed treatment, which could explain this observation. Negative results may have caused a delay in identification and treatment, resulting in additional joint injury (Figure 4).

Table 3 illustrates the distribution of anti-CarP antibodies among male and female participants. In Group 1, 23 out of 35 participants tested positive for all three antibodies: RF, anti-CCP, and anti-CarP antibodies. The remaining 12 cases tested negative for anti-CarP but positive for other antibodies, indicating that the presence of anti-CarP alone is insufficient for diagnosing RA. In Group 2, among the 35 participants who tested negative for both antibodies, 17 tested positive for anti-CarP antibodies. This suggests that the newly identified third antibody can still predict and diagnose RA even when the other two antibodies are absent. Therefore, anti-CarP antibodies play a crucial role in the early diagnosis of RA. In Group 3, out of the 20 patients tested, two were positive for anti-CarP antibodies, aiding in the prediction and diagnosis of RA before the onset of symptoms (Table 3).

**Table 3:** Distribution of anti-carbamylated protein antibodies (biomarkers) between sexes among rheumatoid arthritis and healthy control groups using the chi-square test. This table represents the data in the form of percentages (N1=35, N2=35, N3=20). A p-value less than 0.05 is considered statistically significant; here, the p-value was found to be 0.623, which makes the data statistically insignificant. Anti-CarP: anti-carbamylated protein; RA: rheumatoid arthritis; ns: not significant.

Gender	RA Patients						
	Group 1 N (%)		Group 2 N (%)		Group 3 N (%)		Total N (%)
	Anti-CarP +ve	Anti-CarP -ve	Anti-CarP +ve	Anti-CarP -ve	Anti-CarP+ve	Anti-CarP -ve	
Male	04 (18.2)	05 (22.7)	03 (13.6)	05 (22.7)	0	05 (22.7)	22 (100)
Female	19 (27.9)	07 (10.3)	14 (20.6)	13 (19.1)	02 (2.9)	13 (19.1)	68 (100)
Total	23 (25.6)	12 (13.3)	17 (18.9)	18 (20.0)	02 (2.2)	18 (19.1)	90 (100)
Fisher’s exact test value=3.484, p=.623 (ns)							

**Figure 5:**
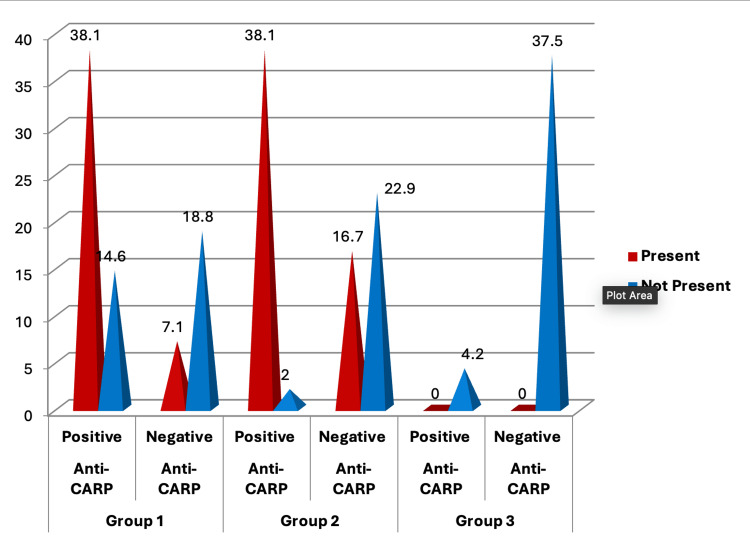
Distribution of anti-carbamylated protein antibodies (biomarkers) in joint damage in rheumatoid arthritis and healthy control groups. This figure represents the data in the form of percentages (N1p=23, N1n=12, N2p=17, N2n=18, N3p=2, N3n=18 where p indicates positivity for the corresponding antibody and n indicates negativity for the same). A p-value less than 0.05 is considered statistically significant; here, the p-value was found to be 0.000, which makes the data statistically significant. Anti-CarP: anti-carbamylated protein.

The link between anti-CarP antibodies and joint injury is depicted in Figure 5. Individuals in Groups 1 and 2 who tested positive for anti-CarP antibodies sustained higher joint injury. This shows that anti-CarP antibodies may cause further joint injury (Figure 5).

**Figure 6:**
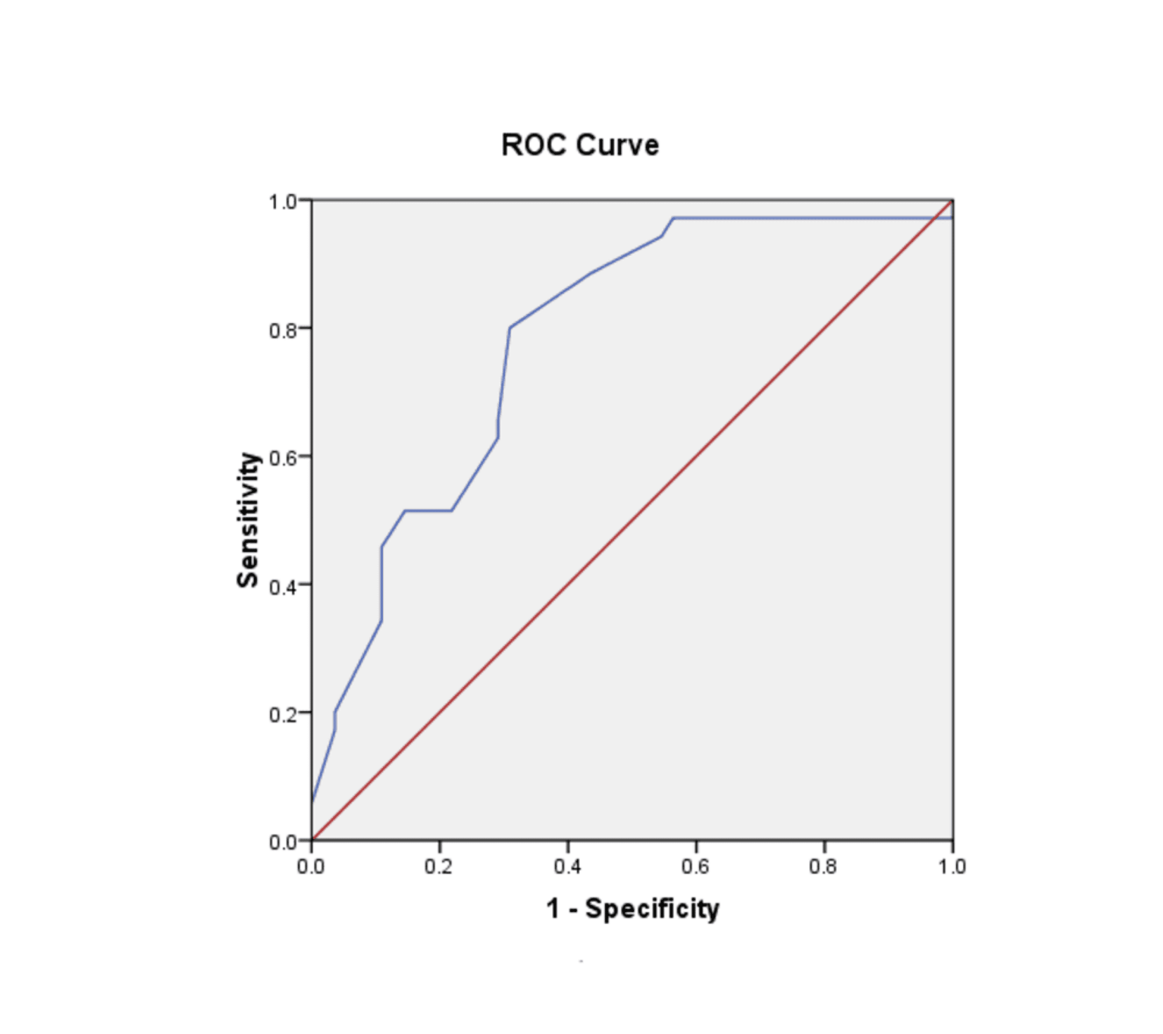
ROC curve for the diagnostic value of anti-CarP antibody for RA. The anti-CarP antibody was tested using ELISA. The cutoff value for positivity was defined using the ROC curve as 4.2 U/mL, with a specificity of 69.1%, a sensitivity of 78.2%, an AUC of 0.782, a 95% CI, and p<0.0001. RA: rheumatoid arthritis; anti-CarP: anti-carbamylated protein; ELISA enzyme-linked immunosorbent assay; ROC: receiver operating characteristic; AUC: area under the curve; CI: confidence interval.

We plotted the receiver operating characteristic (ROC) curve to assess the diagnostic value of anti-CarP antibodies in RA. Our findings revealed a higher specificity of 69.1% for anti-CarP antibodies among individuals with RA, surpassing previous reports. In comparison, RF exhibited a specificity of only 65%, while anti-CCP demonstrated a specificity of approximately 90% in RA patients. However, our study unveiled that anti-CarP antibodies possessed a specificity of 69.1% and a sensitivity of approximately 78.2%. Moreover, this curve yielded a p-value of 0.000, indicating its statistical significance (Figure 6).

Table 4 displays the results of a one-way ANOVA test of anti-CarP, CRP, and ESR levels. The participants in Groups 1 and 2 exhibited significantly higher levels of anti-CarP antibodies compared to the healthy controls in Group 3. Particularly noteworthy, individuals in Group 1 who tested positive for both antibodies displayed anti-CarP levels of approximately 6.63 + 2.23, exceeding those in Group 2. Groups 1 and 2 manifested higher levels of inflammatory markers in comparison to Group 3 (Table 4).

**Table 4:** Comparison of anti-CarP, CRP, and ESR between rheumatoid arthritis patients and healthy controls by Welch’s one-way ANOVA. **This table represents the data in the form of mean ± SD (N1=35, N2=35, N3=20). A p-value less than 0.05 is considered statistically significant; here, the p-value was found to be 0.000, which makes the data statistically significant. Anti-CarP: anti-carbamylated protein; CRP: C-reactive protein; ESR: erythrocyte sedimentation rate.

Parameter	Group 1 N= 35 (Mean ± SD)	Group 2 N=35 (Mean ± SD)	Group 3 N=20 (Mean ± SD)	F-value	Sig**
Anti-CarP	6.63 ± 2.23	4.86 ± 1.6	2.77 ± 1.49	30.699	.000^**^
CRP	35.5 ± 47.46	74.5 ± 53.1	5.3 ± .923	36.252	.000^**^
ESR	36.62 ± 24.52	42.9 ± 20.0	16.1 ± 14.3	17.602	.000^**^

We investigated the association between anti-CarP antibodies and CRP levels across all three groups. A moderate positive correlation was observed between the healthy group (Group 3) and the RF and anti-CCP-positive group (Group 1) (r = 0.444, p = 0.001). This correlation was linear in Group 1, showing no discernible positive or negative correlations, with an R-squared value of 0.068. Additionally, a moderate positive relationship was found between anti-CarP antibodies and ESR in both the healthy group (Group 3) and the RF and anti-CCP-positive group (Group 1) (r = 0.438, p = 0.001). Furthermore, a stronger positive association was identified between anti-CarP antibodies and CRP levels in the healthy group (Group 3) and the RF and anti-CCP-negative group (Group 2). As the level of anti-CarP antibodies increased, so did the CRP level (r = 0.094), a statistically significant finding. Similarly, there was a positive correlation between anti-CarP antibodies and ESR in both the healthy group (Group 3) and the RF and anti-CCP-negative group (Group 2) (r = 0.529, p = 0.001). As the amount of anti-CarP antibodies increased, so did the ESR, with a statistically significant difference (p = 0.001). In summary, moderate positive relationships were observed between anti-CarP antibodies and CRP or ESR levels across all three groups. The correlations were linear in Group 1 and positive in Groups 2 and 3. Notably, as the level of anti-CarP antibodies increased, both CRP and ESR readings also increased significantly.

## Discussion

Our study identified the presence of anti-CarP antibodies in RA patients, consistent with previous research [18]. Among the 70 RA patients examined, 53 were middle-aged women. Yee et al. found that 30% of RA patients tested positive for anti-CarP antibodies [18], while Othman et al. reported a 41.9% positivity rate [19,20]. In our study, approximately 57.1% of RA patients tested positive for anti-CarP antibodies. In Group 1 (RF and anti-CCP positive), 23 out of 35 individuals tested positive for anti-CarP, accounting for 65.7%. This substantial proportion suggests that anti-CarP can serve as a biomarker alongside the other two antibodies for identifying RA. In Group 2, among the 35 RA subjects (RF and anti-CCP negative), 17 were positive for anti-CarP, representing approximately 48.5%. This indicates that even when the other two antibodies are negative in an RA patient, anti-CarP can still be detected. The utility of anti-CarP positivity in individuals who are anti-CCP and RF-negative is valuable for identifying RA.

Previous research has indicated that anti-CarP antibodies can be detected up to a decade before symptom onset, facilitating early intervention to prevent disease progression and joint damage. Anti-CarP antibodies play a crucial role in averting joint injury, persisting in healthy individuals for several years before the onset of RA symptoms. In our investigation, two healthy individuals tested positive for anti-CarP antibodies. Interestingly, one of these individuals had a family history of arthritis, while another reported experiencing joint discomfort, suggesting an increased risk of developing RA soon. Such individuals should undergo regular screenings to facilitate early detection and prompt treatment.

Our study uncovered a significant disparity in anti-CarP antibody levels between healthy individuals and those with RA, consistent with prior studies [21]. Among the 40 RA individuals tested for anti-CarP antibodies, 32 exhibited notable joint damage compared to anti-CarP antibody-negative individuals. This aligns with Humphreys et al.'s 2015 study, which found increased disability and disease activity in polyarthritis patients who tested positive for anti-CarP antibodies [21-23]. These findings suggest that the presence of anti-CarP antibodies can predict underlying chronic RA and enable the diagnosis of joint damage much earlier than other antibodies. CRP has served as a longstanding marker for over 80 years in diagnosing inflammatory disorders such as RA. Numerous studies have established a robust association between CRP and disability levels, corroborating our findings. Our study unveiled a statistically significant association between joint damage and CRP levels, as well as a positive correlation between CRP and anti-CarP antibodies, indicating their interconnectedness.

According to prior research, the specificity of RF for RA is relatively low, approximately 65%, as RF can also be present in other autoimmune disorders. In our study, the sensitivity of anti-CarP was approximately 69.1%, significantly higher than that of RF alone. Additionally, the sensitivity of the anti-CarP antibody was approximately 78.1%. Consequently, alongside RF and anti-CCP antibodies, anti-CarP antibodies may serve as diagnostic markers for RA. Anti-CarP antibodies represent promising new biomarkers for the diagnosis of RA. However, a limitation of our study is that the follow-up duration was not sufficiently long to establish a correlation between anti-CarP antibodies and disease progression or activity/extra-articular involvement in RA patients.

## Conclusions

In conclusion, our study underscores the potential of anti-CarP as a valuable biomarker in the context of RA. With moderately high specificity and sensitivity, anti-CarP antibodies demonstrated significant promise in aiding RA diagnosis and prognosis. Particularly noteworthy is their correlation with CRP levels and association with severe joint damage, suggesting their utility in monitoring disease progression. The identification of anti-CarP positivity in a substantial portion of RA patients who are negative for both anti-CCP and RF highlights its value in diagnosing RA, particularly in cases where conventional biomarkers fail to yield definitive results. Furthermore, the presence of anti-CarP antibodies in approximately 10% of healthy controls underscores their potential as an early predictor of RA, aligning with prior research indicating their detectability up to a decade before symptom onset. Although a notable proportion of patients tested negative for anti-CarP in our study, the combination of anti-CarP with RF and anti-CCP holds promise for enhancing the diagnostic accuracy of RA. Overall, our findings advocate for the inclusion of anti-CarP antibodies in the diagnostic arsenal for RA, facilitating comprehensive early detection and effective management of this debilitating condition.
